# Minimizing Mistakes and Embracing Uncertainty

**DOI:** 10.1371/journal.pmed.0020272

**Published:** 2005-08-30

**Authors:** The PLoS Medicine Editors

## Abstract

Scientific truth is a moving target. Journals must apply rigorous standards, but publication of data and conclusions before they are proven true is part of the research enterprise.

*“Truth in science can be defined as the working hypothesis best suited to open the way to the next better one.”—Konrad Lorenz, Austria*

Scientific truth is a moving target. In the process of peer review, authors, reviewers, and editors work together to minimize the reporting of false results. However, even if one assumes no bias, wrongdoing, or ignorance on the part of any of the individuals involved—which is unrealistic, no doubt—chances are that some findings will turn out to be false. But is it inevitable, as John Ioannidis argues in an Essay in this issue of *PLoS Medicine* (DOI: 10.1371/journal.pmed.0020124), that the majority of findings are actually false?

Although his calculations are based on assumptions about complex scenarios that we do not fully understand—as is true for most research projects—Ioannidis argues convincingly that many published findings will turn out to be false.

Ioannidis is not the first to raise some of these concerns. Indeed, there are already initiatives under way that seek to address them. Increasingly, researchers design individual studies, systematic reviews, and meta-analyses using Bayesian statistics, in which the issue of pre-study odds is taken into account. And issues such as reducing sources of bias when assessing evidence are addressed in the methodology used by the Cochrane Collaboration in the production of its systematic reviews.

Ioannidis doesn't define “findings,” but it seems important to attempt to separate data (“in this study 5% of people examined who lived in San Francisco from 1965–1970 developed lung cancer compared with 20% of people studied who lived in Anchorage”) from conclusions (“lung cancer rates are higher in Anchorage than San Francisco”) and hypotheses (“cold weather exacerbates the consequences of smoking”).

Hypotheses will inevitably change, as they depend not only on the study but also on the context of other relevant research and knowledge. Conclusions are also often based on current knowledge and assumptions, and, thus, subject to change. The data should be more robust; for instance, other researchers applying the same methods to study the same group of patients at the same time should be able to generate the same data. However, research progress depends on conclusions being tested elsewhere. The major issue about the truth of research findings would therefore seem to concern the conclusions, and Ioannidis's claim that most conclusions are false is probably correct. Is that a problem? Can it be avoided?

The possibility that most conclusions are false might be an inevitable part of the research endeavor. That said, researchers and those involved in publication of research must do what they can to reduce false conclusions.

One way to do this is to delay publication until such a time when the chances that a conclusion is true are sufficiently high. If many published conclusions are false, we (editors and reviewers) need to ask ourselves whether we are setting the bar too low. But what is the consequence of setting it higher?

Research progress depends on dissemination of results, and journal articles are the most effective tool we currently have to share them. The answer, therefore, cannot be that we wait until conclusions are proven beyond a doubt before we publish them. Publication of preliminary findings, negative studies, confirmations, and refutations is an essential part of the process of getting closer to the truth. Everyone involved in the generation and publication of research results needs to be open-minded, rigorous, and honest in designing experiments, analyzing results, reporting findings, peer-reviewing manuscripts, providing comments, and accepting that uncertainty exists in research.

Ioannidis suggests how studies could be designed from the outset to increase their chances of producing true results. He also gives some corollaries that allow readers to get a sense of the extent of uncertainty for a particular study. He stresses that reliable evidence generally comes from several studies and from several teams of researchers, and that what matters is the totality of the evidence.

What can editors do? At high-impact journals such as *PLoS Medicine*, we see it as our job to select important articles. This means the conclusions reported should be more rather than less likely to be true. But better measures of importance are that a study should address a substantial clinical or public- health question, in as rigorous a way as possible, and the findings should be likely to have an effect on how other researchers think about the question. In reporting studies, we ask that data are clearly delineated from conclusions, and conclusions from hypotheses. In addition to individual studies, editors should (and at *PLoS Medicine* we do) ensure there is a place for articles that synthesize evidence from different sources.

Too often editors and reviewers reward only the cleanest results and the most straightforward conclusions. At *PLoS Medicine*, we seek to create a publication environment that is comfortable with uncertainty. We encourage authors to discuss biases, study limitations, and potential confounding factors. We acknowledge that most studies published should be viewed as hypothesis-generating, rather than conclusive. And we publish high-quality negative and confirmatory studies.

We also accept some responsibility for educating consumers of research about the research process. Consumers also need to become comfortable with uncertainty, and understand the strengths and weaknesses intrinsic to every study conducted and published. Besides selecting papers and influencing how results are reported, we use the synopses and patient summaries to highlight uncertainties in research papers. We also encourage contributions such as the essay by Ioannidis to our magazine section that will help research producers and consumers to understand research findings in context.

